# Addressing Data Quality Challenges in Lung Cancer Data Within the Observational Medical Outcomes Partnership Common Data Model: Observational Study

**DOI:** 10.2196/90246

**Published:** 2026-06-08

**Authors:** Jens Declerck, Mieke Deschepper, Kirsten Colpaert, Dipak Kalra, Pascal Coorevits

**Affiliations:** 1 Department of Public Health and Primary Care, Ghent University, Unit of Medical Informatics and Statistics, Corneel Heymanslaan 10, Ghent, Belgium, 32 0474538199; 2 The European Institute for Innovation through Health Data, Ghent, Belgium; 3 Ghent University Hospital, Data Science Institute, Ghent, Belgium

**Keywords:** health data quality, Observational Medical Outcomes Partnership Common Data Model, OMOP CDM, primary use, secondary use, extract, transform, and load, ETL

## Abstract

**Background:**

The secondary use of health data is essential for advancing medical research and improving clinical practice. The Observational Medical Outcomes Partnership (OMOP) Common Data Model (CDM) enables large-scale, multicenter studies but faces challenges related to consistency, completeness, and transparency during data mapping from original data sources.

**Objective:**

This study aimed to evaluate the quality of the mapping process for lung cancer data within the Federated Health Innovation Network project, with a focus on consistency, completeness, and challenges encountered throughout the process.

**Methods:**

Clinical data from Ghent University Hospital were mapped to the OMOP CDM using a reference data dictionary. Consistency was assessed using Cohen kappa (κ) scores, while completeness was evaluated by comparing patient and record counts before and after mapping. Challenges, including unstructured data and an evolving reference standard, were documented and analyzed.

**Results:**

High consistency was observed for structured variables, while some unstructured variables, such as “Smoking status,” were excluded due to their free-text format and the lack of suitable OMOP concepts. The completeness analysis showed minimal data loss for most structured variables but highlighted substantial challenges associated with unstructured data. Persistent issues included evolving data dictionary versions and mismatches in diagnostic code granularity between institutions, underscoring structural challenges in standardization.

**Conclusions:**

The transformation of lung cancer data to the OMOP CDM highlighted both technical and systemic challenges, including the handling of unstructured data and the resolution of granularity discrepancies. A multidisciplinary approach involving clinical and technical expertise is crucial for ensuring reliable, high-quality datasets for multicenter research.

## Introduction

The secondary use of health data—leveraging existing health information for purposes beyond direct patient care—has become a cornerstone for advancing medical research [1,2], developing health care policies [3,4], and improving clinical practices [5]. By integrating health data from diverse clinical settings, researchers can uncover valuable insights into disease patterns [6], treatment outcomes [7], and health care processes [8]. This approach is particularly crucial for studying rare diseases or uncommon clinical events, in which data from a single source are often insufficient [9]. The power of large-scale, multicenter datasets lies in their ability to address complex research questions, but this potential can only be fully realized if data quality is ensured throughout the entire data lifecycle—from primary data capture to transformation and integration into a standardized framework for secondary use [10,11].

Data quality remains one of the most substantial challenges in the effective secondary use of health data [11,12]. Poor-quality data can lead to incorrect research findings [13], poor clinical decision-making [14], and misguided health care policies [15]. Data quality is influenced by multiple factors, including the reliability of the primary data sources, the transformation processes used to standardize data, and the quality of the resulting secondary datasets [11]. The extract, transform, and load (ETL) process plays a critical role, as it involves consolidating, standardizing, and integrating data from multiple sources. Each stage of the ETL process presents unique challenges and risks to data quality. Errors at any stage can compromise the usability and reliability of the final dataset, leading to potential misinterpretations in downstream analyses [16,17].

Although frameworks addressing data quality in primary and secondary datasets are well established [11,18-22], mapping clinical data from different sources into a standardized model such as the Observational Medical Outcomes Partnership (OMOP) Common Data Model (CDM) remains prone to challenges [4]. The OMOP CDM is a standardized framework for structuring and analyzing health care data from diverse sources, such as electronic health records (EHRs). By adopting uniform data structures and standardized terminologies, such as Systematized Nomenclature of Medicine–Clinical Terms (SNOMED CT), Logical Observation Identifiers Names and Codes (LOINC), and *International Classification of Diseases, 10th Revision* (*ICD-10*), the OMOP CDM facilitates interoperability, collaborative research, and large-scale data analysis. OMOP provides a robust framework for integrating diverse datasets by standardizing both data structure and terminologies [3], thereby enabling multicenter research on treatment outcomes [23] and health care delivery [24]. However, variability in data extraction and mapping practices can introduce inconsistencies, thereby affecting the reliability and reproducibility of research findings [25].

Despite the increasing adoption of the OMOP CDM and the growing usability of secondary datasets [26], there is limited guidance on how to systematically evaluate mapping quality or address discrepancies in granularity and completeness [27]. This lack of structured approaches for assessing the transformation process creates barriers to new implementations, particularly in multicenter settings.

This study sought to address these gaps by focusing on the quality of the mapping process during the implementation of the OMOP CDM for lung cancer data. This effort was part of the Federated Health Innovation Network (FHIN) project, an open-source collaboration among Belgian hospitals to develop a fully automated, federated platform aimed at addressing research questions in the field of lung cancer [28]. As part of this project, a data dictionary was provided, detailing the mapping of raw data to OMOP CDM concepts. This dictionary, which outlined one-to-one relationships between raw data elements and OMOP CDM concept IDs, served as a reference standard.

Specifically, this study aimed to explore strategies for preserving data quality during the process of mapping data to the OMOP CDM. The primary objective was to evaluate the quality of the mapping process by examining the completeness and consistency during the mapping process. The secondary objective was to identify the challenges and complexities encountered during the implementation of the OMOP CDM and to develop a practical framework to guide future OMOP implementations.

## Methods

### Study Design and Setting

This study was independently conducted by the Data Science Institute (DSI) of Ghent University Hospital and the European Institute for Innovation through Health Data to ensure transparency and traceability of the mapping process of clinical data into the OMOP CDM. This study evaluated the mapping of clinical data to the OMOP CDM within the context of lung cancer data integration, with a focus on reproducibility and data quality assessment. Although not part of the FHIN project, this study aligns with its goals by ensuring rigorous documentation and standardization of the mapping process for lung cancer data. The study design emphasizes reproducibility and adaptability to similar multicenter initiatives.

### Ethical Considerations

This study did not undergo a formal institutional review board or research ethics committee assessment because it was based on fully anonymized data and did not involve direct interaction with human participants. No identifiable personal data were accessed, and all data were handled in compliance with applicable data protection regulations.

### Reference Standard Provided by the FHIN Project

As part of the FHIN project, a data dictionary was provided that defined key data items relevant to lung cancer and their mapping to specific OMOP concept IDs. This dictionary served as the reference standard for evaluating the consistency of our mapping process. Examples of some of the variables included in the data dictionary are provided in Table 1. However, the dictionary lacked critical details, including the original data sources for the variables, the extraction logic (eg, identification of the relevant tables and fields for each data element and transformation of field values to the standard terminology relevant in OMOP), and the rationale behind assigning specific concept IDs. Additionally, the data dictionary evolved throughout the project, reflecting adjustments made as part of the data quality process. These factors complicated efforts to fully replicate the mapping process, potentially introducing variability and bias. For transparency, we based our evaluation on the version of the data dictionary available as of December 1, 2024.

**Table 1.** Example of the data dictionary.

Concept ID	Concept name	Vocabulary ID	Concept code	Observational Medical Outcomes Partnership table
44790293	Radiotherapy delivery	SNOMED CT^a^	231711000000108	PROCEDURE
40483776	Total radiation dose delivered	SNOMED CT	445461008	MEASUREMENT
4155148	Delivered radiation dose	SNOMED CT	371892002	MEASUREMENT

a SNOMED CT: Systematized Nomenclature of Medicine–Clinical Terms.

### Data Quality Assurance

Data quality assurance was performed to evaluate completeness and consistency. Completeness was assessed by comparing the total number of patients and records extracted from the raw data sources with those successfully transformed into OMOP CDM tables. Consistency assessment was performed to evaluate the agreement between the OMOP concept IDs assigned during the ETL process and those specified in the reference data dictionary. For each data category, which typically included multiple variables, Cohen κ scores [29] were calculated at the variable level. Agreement was defined as an exact match between the concept ID assigned during mapping and the expected concept ID in the dictionary. Variables that were unmapped, mismatched, or lacked a valid concept ID were considered disagreements. To report a single consistency score per category, the final κ score was calculated as the unweighted average of the individual κ scores of all variables within that category, as a descriptive summary measure across heterogeneous variables [30]. To capture within-category variability, the SDs of the κ scores and the number of variables per category were additionally calculated and reported. This approach allowed a balanced assessment across categories, independent of variable count or complexity, and helped identify specific areas of misalignment in the mapping process.

### Data Sources and Extraction

On the basis of the variables defined in the data dictionary, all relevant data items were extracted from the data sources at Ghent University Hospital. The extracted data included records from patients between January 1, 2016, and December 31, 2023. The extraction process involved developing and executing SQL queries to retrieve the specified variables from various hospital databases. These data sources included the DSI–Data Warehouse (DWH); Multidisciplinary Oncology Consultation (MOC) application; *Minimale Ziekenhuisgegevens* (MZG); General Laboratory Information Management System; and admission, discharge, and transfer systems, each containing data relevant to the mapping process. The DSI-DWH is a curated database where vital parameters, such as weight and height, are stored. Data with the same meaning (eg, weight) were extracted from different fields within the EHR, cleaned, and standardized. MOC application [31] provides essential histology and pathology information, particularly related to cancer cases. MZG [32] stores diagnostic codes in *ICD-10* format, which are key for mapping clinical diagnoses. The General Laboratory Information Management System contains analysis codes and laboratory values classified using LOINC. Finally, the admission, discharge, and transfer system contains administrative variables and additional patient characteristics.

The data were stored in a staging area with tables reflecting the structure of the source systems. This staging area enabled uniform querying across databases, ensuring that data were harmonized before the ETL process. By implementing a structured extraction workflow, errors were minimized, and traceability from source to target was ensured.

### ETL Process

The ETL process was implemented to harmonize extracted data into OMOP CDM version 5.4. During extraction, raw data were stored in a centralized Microsoft SQL database for processing. Transformation involved automated and manual mappings to OMOP standards. Automated mappings were conducted with SQL scripts using the OMOP vocabularies. An example of the mapping from *ICD-10* to standard OMOP codes can be found in Multimedia Appendix 1. This process aligned source data with terminologies such as SNOMED CT, LOINC, and *ICD-10*. Manual mappings were facilitated by Keun [33], particularly for complex variables such as genetic mutations; tumor, node, and metastasis (TNM) staging; and World Health Organization functional scores. SQL scripts were developed to transform raw data into OMOP-compliant tables. These scripts ensured that variables were assigned to appropriate domains and that data transformations adhered to OMOP guidelines. Special attention was given to the handling of unstructured data, such as free-text variables, which posed challenges during mapping. The final load process was executed using the FHIN tool Rabbit-in-a-Blender [34], an ETL pipeline used to transform raw data into the OMOP CDM. Mapping approaches varied by data category, with both automated and manual strategies applied. Manual mapping was used for categories such as “Clinical TNM staging,” “Pathological TNM staging,” and “Genetic mutations.” Automated mapping was applied to standardized classifications, including “Histology,” “Laboratory tests,” and “Diagnosis.”

## Results

### Consistency

The mapping process involved 12 data categories necessary for transforming lung cancer data into the OMOP CDM. These categories included essential clinical, genetic, and demographic variables such as diagnostic codes, TNM staging, and genetic mutations (eg, Kirsten rat sarcoma and v-raf murine sarcoma viral oncogene homolog B). Mapping methods varied by category, using either manual processes requiring domain expertise or automated methods for which established standards enabled straightforward mapping. Manual mapping was used for categories such as “Clinical TNM staging,” “Pathological TNM staging,” and “Genetic mutations,” where specialized knowledge was critical to ensure accuracy. Automated mapping was applied to standardized classifications, including “Histology” (based on the International Classification of Diseases for Oncology 3rd edition classification), “Laboratory tests” (based on the LOINC classification), and “Diagnosis” (based on the *ICD-10* classification). Additional details can be found in Multimedia Appendix 1.

To evaluate mapping consistency, κ scores were calculated at the variable level within each data category by comparing assigned OMOP concept IDs with those defined in the reference data dictionary. For each category, the reported κ represents the unweighted mean of the variable-level κ values. In addition, the number of variables per category and the SD were calculated to reflect variability within categories. A mean score of 1 indicates perfect alignment across all variables in that category, while lower scores and higher SD values highlight categories in which mapping challenges or heterogeneity were more pronounced. The mean κ score, SDs, and number of variables per category are presented in (Table 2).

**Table 2.** Mean kappa (κ) scores, SDs, and number of variables per category according to the mapping process.

Mapping processes and categories	Variables, n (%)	Cohen κ score, mean (SD)	
Automated mapping			
Diagnosis	19 (5.3)	0.842 (0.375)	
Histology	26 (7.3)	1.000 (0)	
Laboratory tests	185 (52)	0.968 (0.178)	
Manual mapping			
Clinical TNM^a^ staging	38 (10.7)	0.921 (0.273)	
Gender	2 (0.6)	0 (0)	
Genetic mutations	4 (1.1)	0.500 (0.577)	
Pathological TNM staging	27 (7.6)	0.926 (0.267)	
Smoking status	1 (0.3)	0 (N/A^b^)	
Therapy procedures	3 (0.8)	0.333 (0.577)	
Unit	43 (12.1)	0.372 (0.489)	
Value	2 (0.6)	1.000 (0)	
World Health Organization score	6 (1.7)	0.833 (0.408)	

a TNM: tumor, node, and metastasis.

b N/A: not available.

High consistency was observed in most categories. Categories such as “Value” and “Histology” showed perfect agreement (mean κ 1.000, SD 0), indicating fully consistent mappings across all variables within these categories. “Laboratory tests” and “Diagnosis” demonstrated strong agreement (mean κ 0.968, SD 0.178 and mean κ 0.842, SD 0.375, respectively), with variability observed at the variable level.

Both “Clinical TNM staging” and “Pathological TNM staging” showed high mean κ values (0.921 and 0.926, respectively) but with notable SDs (0.273 and 0.67, respectively), indicating variability across individual variables. Categories such as “Unit” (mean κ 0.372, SD 0.489) and “Therapy procedures” (mean κ 0.333, SD 0.577) exhibited lower consistency and substantial variability, driven by differences in unit recording practices and the absence of specific OMOP concept IDs for certain therapies (eg, immunotherapy).

“Gender” and “Smoking status” showed no agreement (κ=0). For “Smoking status,” no variability measure could be calculated (n=1), reflecting complete mapping failure due to unstructured source data. For “Gender,” the κ value of 0 was due to the absence of this variable in the reference standard. Consequently, no predefined mapping specification was available, leading to a mismatch between the implemented mapping and the expected reference standard. These findings indicate variability in the data dictionary and the lack of a structured representation of specific variables within the source systems.

### Completeness

Completeness was assessed by comparing the number of records and patients before and after mapping. The initial data quality test revealed that only approximately half of the patients were successfully mapped. This low completeness rate was associated with discrepancies in *ICD-10* code mappings and inconsistencies across source systems. For example, some patients diagnosed with lung cancer appeared in the MZG data source but were missing from the MOC application data, or vice versa. These mismatches were accompanied by the temporary exclusion of affected records from the mapped dataset. An iterative data quality improvement process was applied. With each iteration, additional patients were reincluded. By the final iteration, only a single patient remained excluded due to an unresolved classification issue. This patient had relevant clinical data in the laboratory system but was categorized in the source data as an outpatient consultation. As the ETL pipeline was configured to extract only hospitalized patients, this record could not be incorporated. Consequently, all but one patient were successfully included in the final dataset.

For most variables, patient and record counts remained complete in the final dataset, indicating a successful transformation. However, a few exceptions persisted. The variable “Smoking status” exhibited complete data loss because it was stored in an unstructured free-text field that combined information on smoking, drug abuse, and alcohol use. This format made it impossible to extract smoking-specific content for standardized mapping. Additionally, minor data loss was observed in the “Unit” and “Diagnosis” categories, which stemmed from inconsistencies in data representation or mapping complexity.

### Challenges Encountered During the Mapping Process

**Figure 1.**
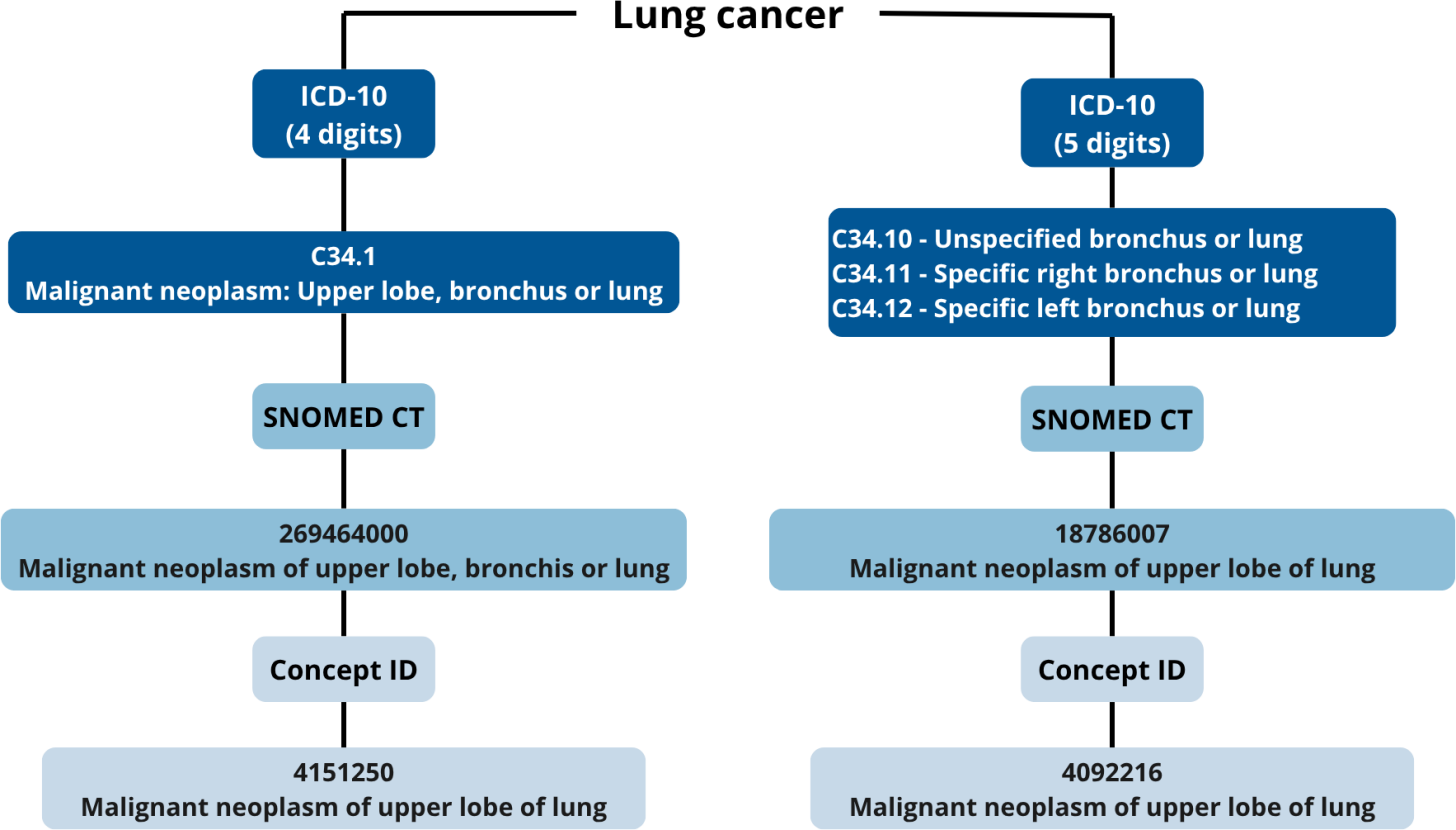
Different Systematized Nomenclature of Medicine–Clinical Terms (SNOMED CT) codes derived from code C34.1. *ICD-10*: *International Classification of Diseases, 10th Revision*.

Implementing the OMOP CDM revealed several structural and semantic challenges that complicated the mapping process. One considerable issue was the difference in data granularity between our hospital and the reference standard. For instance, in the condition table, the *ICD-10* code C34.1, which represents lung cancer of the upper lobe, was inconsistently mapped to different OMOP codes based on whether a 4-digit (C34.1) or 5-digit (C34.10) variation of the code was used. Although there is no clinical difference between codes C34.1 and C34.10, this inconsistency arose because the data dictionary only accounted for 4-digit codes, whereas the system at our hospital used a more granular collection process. Furthermore, SNOMED CT, the standard OMOP vocabulary for conditions, introduced additional complexity by mapping back to multiple codes for a single condition. This duality made maintaining consistency and alignment with the OMOP CDM challenging. This is presented in Figure 1.

Another major complication was the reliance on free-text fields in the source data. For instance, the “Smoking status” variable was captured in an unstructured field in the EHR that combined drug abuse, smoking, and alcohol abuse into a single text box. Consequently, it was impossible to reliably determine whether the recorded information referred specifically to smoking, alcohol use, or drug abuse. This unstructured format prevented mapping to standardized OMOP concepts and led to the exclusion of these data during transformation. Notably, no neuro-linguistic programming techniques were used in this research, further limiting the ability to computationally extract and interpret such information. These issues were further exacerbated by the design of our hospital’s EHR system, which is a home-grown platform historically optimized for clinical documentation rather than structured data capture. The current configuration of the EHR, with limited use of standardized fields, made the extraction and transformation process more challenging. Height and weight were initially derived from unstructured data fields in the EHR. However, a curation and parsing workflow was established, making these variables usable within the project (as stored in the DSI-DWH). A transition toward more structured data entry has recently been initiated, which is expected to facilitate future data standardization.

A more subtle but impactful challenge was the evolving state of the data dictionary during the project. As no finalized version was agreed upon at the project’s start, the data dictionary continued to evolve, often introducing inconsistencies. For this study, we used the version of the dictionary established on December 1, 2024. However, several updates (such as the change in the source of chemotherapy data from procedures to medication records) required periodic reassessment of our mappings. Although these changes were ultimately controlled for, they contributed to mapping delays and highlighted the need for stable definitions early in such projects.

Beyond data structure and documentation, the specialized knowledge required for OMOP CDM mapping proved to be a limiting factor. Mapping variables, resolving inconsistencies, and applying the correct logic demanded not only a solid understanding of the OMOP CDM but also clinical insight into the source data. The lack of domain expertise within the team sometimes caused delays, especially when clinical interpretation was needed to resolve ambiguous cases.

Finally, inconsistencies across the OMOP projects themselves presented challenges. Interactions with other OMOP initiatives revealed differences in data dictionaries and coding approaches. These differences included variations in variable definitions, mapping choices, and levels of coding granularity for similar clinical concepts. Consequently, alignment across projects required additional reconciliation efforts, increasing the complexity of the mapping process.

## Discussion

### Principal Findings

This study evaluated the quality of the mapping process of lung cancer data to the OMOP CDM, with a focus on completeness and consistency, and identified key challenges encountered during implementation. Overall, high consistency was achieved for structured variables, while unstructured data and variability in coding practices posed challenges. Completeness improved substantially through iterative data quality refinement, highlighting the importance of continuous validation during the ETL process. These findings align with the study objectives of assessing mapping quality and identifying barriers to effective OMOP implementation.

### Data Quality Assessment

The findings demonstrated variability in consistency and completeness across categories. Variables with structured data and robust reference standards, such as “Histology,” “Value,” and “Laboratory tests,” achieved high κ scores with low variability, indicating stable and reproducible mappings across variables. In contrast, categories that required greater clinical interpretation or manual mapping, including “Clinical TNM staging,” “Pathological TNM staging,” and “Genetic mutations,” showed high κ values but substantial SDs, reflecting heterogeneous agreement at the variable level. This variability indicates that although overall mapping performance was strong, individual variables within these categories posed specific challenges.

A key contributor to this variability was differences in coding granularity. Variations in the level of detail captured in source systems, such as the use of 4-digit vs 5-digit *ICD-10* codes, introduced inconsistencies during mapping despite representing clinically equivalent concepts. This reflects a broader challenge in OMOP ETL processes, in which differences in coding specificity across institutions can affect semantic alignment and the consistency of standardized data.

Categories such as “Unit,” “Therapy procedures,” “Gender,” and “Smoking status” encountered challenges, reflecting the difficulties associated with ambiguous, incomplete, or unstructured data. These findings were consistent with previous studies, in which effective mappings are established for well-structured and standardized variables [35]. Unstructured variables, such as “Smoking status,” posed a particular challenge. Captured as free text in the EHR, this field combined multiple categories, making it impossible to extract smoking-specific information for mapping to OMOP concepts. These findings highlight a challenge in OMOP implementations related to the handling of unstructured data, which requires additional preprocessing before integration into the standardized model. Previous research has shown that free-text data often leads to data exclusion during OMOP transformation, limiting the accuracy of analyses reliant on such variables [3,4,36].

The completeness analysis revealed that structured data generally retained patient and record counts after mapping. For instance, variables such as “Histology” and “Radiotherapy” achieved nearly complete preservation of records. However, the absence of structured standards for certain categories led to minor data loss. For example, inconsistencies in the recording of units and granularity differences in diagnostic codes resulted in missing data during transformation. Although these losses were minimal, they highlight the need for enhanced preprocessing and harmonization workflows to mitigate discrepancies across source systems. These findings align with previous research that has identified similar challenges [4,35].

### Challenges Encountered

The transformation process of mapping lung cancer data to the OMOP CDM highlights several challenges, encompassing both technical data issues and broader systemic and knowledge-related barriers. Although the technical aspects of the data, such as unstructured text and inconsistent coding practices, are well-recognized sources of data quality issues [37], this study demonstrates that the challenges extend beyond these technical constraints.

The major challenge encountered was related to the data dictionary. This data dictionary, provided by the reference hospital, offered a 1-to-1 mapping between raw data elements and OMOP concept IDs, serving as a useful starting point. However, 2 challenges emerged related to the data dictionary. First, frequent updates by the reference hospital invalidated previously consistent mappings, forcing manual remapping efforts. This not only increased the workload but also introduced a higher risk of mapping errors. These disruptions highlight the critical need to finalize a stable and harmonized data dictionary before initiating the project. Establishing such a standardized data dictionary would minimize unnecessary adjustments and reduce inconsistencies during the mapping process. Second, although the 1-to-1 mapping approach provided by the reference site was initially helpful, the transformation process revealed its limitations. A more enriched data dictionary is essential to support consistent and complete mappings. This enriched version should include detailed mapping rules, clinical context, and clear rationales for assigning raw data elements to specific OMOP IDs to address these gaps.

Another challenge lies in the variability of how data are collected, structured, and recorded across hospitals. Unstructured data, such as free-text entries in EHRs, often result in missing or unusable data during mapping [38]. Similarly, coding discrepancies, such as differences in granularity between source data, can lead to inconsistencies and missing values [39]. These technical issues not only reduce the completeness of the mapped data but also hinder its clinical applicability and analytical utility. Differences in granularity, terminology, and data format between hospitals further exacerbate these challenges, introducing biases during the extraction and mapping process.

Beyond technical challenges, the success of the transformation process depends heavily on the knowledge and expertise of the individuals involved. Mapping requires a deep understanding of the OMOP CDM framework, including its vocabularies, as well as comprehensive knowledge of the clinical and technical aspects of the source data and source systems. Insufficient expertise can result in mapping errors or inconsistencies, particularly for complex variables requiring nuanced interpretation. Differences in how data are collected and structured between the 2 hospitals introduced inconsistencies in the mapping process. Differences in source systems between institutions can introduce biases, as variations in data collection may not always be fully accounted for in the mapping strategy.

### Implications for Practice and Research

The findings of this study have several implications for future OMOP implementations. First, they highlight the importance of structured data capture at the source, as unstructured data limits downstream usability. Second, there is a need for stable and enriched data dictionaries that include detailed mapping logic and clinical context. Finally, the results demonstrate that iterative data quality assessment is essential to achieve high completeness and consistency.

Mapping raw health data to the OMOP CDM is a complex process requiring in-depth planning, a structured approach, and multidisciplinary collaboration to ensure high-quality outcomes. On the basis of the insights from this study, the following recommendations are proposed to address gaps identified during the transformation process:

1. Develop an enriched and stable data dictionary: move beyond one-to-one mappings by creating a data dictionary that includes detailed mapping logic and explanatory rationale. This enriched data dictionary should capture the clinical context of variables (eg, the underlying reason and circumstances under which a variable is captured), the structure of the source data, and any transformations applied to align with OMOP conventions. Finalizing a stable and harmonized data dictionary before initiating the project will prevent data quality issues from occurring during the transformation process.
2. Leverage data profiling to address variability across hospitals: systematically profile source data to understand their structure, coding practices, completeness, and variability. This includes identifying differences in data formats, coding systems (eg, *ICD-10* use), value distributions, and missing data patterns prior to mapping.
3. Strengthen expertise through training and collaboration: equip mapping teams with training programs that provide an in-depth understanding of the OMOP CDM framework, including its tables, relationships, and vocabularies [40]. Encourage collaboration between medical and technical experts to ensure that mapping strategies capture both clinical accuracy and technical precision.
4. Automate the mapping process where possible: automate the mapping process where feasible to improve consistency and reduce manual effort, particularly for standardized variables.
5. Conduct data quality assessments: perform data quality assessments before and after mapping to identify inconsistencies and ensure completeness of the transformed dataset.

These recommendations provide a structured framework to improve mapping quality and enhance the reliability of standardized datasets for multicenter research.

### Limitations

This study has some limitations. First, no formal data quality assessment was performed after extracting raw data. Consequently, potential inaccuracies or inconsistencies in the source data may have influenced the mapping outcomes, affecting the quality of the final OMOP dataset. Second, the role and timing of medical expert involvement remain unclear. Although their expertise is crucial for interpreting raw data and ensuring consistent mappings, their absence during the creation of the data dictionary may have limited its clinical relevance. Finally, uncertainty about when to involve experts in the workflow—whether during data interpretation, technical mapping, or both—may have affected the consistency of the process. Addressing these limitations through postextraction quality checks and clearer integration of medical expertise can improve the trustworthiness and reliability of future mapping efforts.

### Conclusions

This study highlights the challenges of mapping lung cancer data to the OMOP CDM, particularly in managing unstructured data, addressing granularity discrepancies, and adapting to evolving reference standards. Although high consistency was achieved for structured variables, limitations in handling free-text data and incomplete mapping logic documentation revealed areas for improvement. The interplay between technical challenges and nontechnical factors, such as human expertise and system variability, highlights the need for a multidisciplinary approach to OMOP CDM implementation. Collaborations between clinical experts, data scientists, and data engineers are essential to bridge gaps in knowledge and address the complexities of transforming diverse health care data into a standardized format. Furthermore, fostering a shared understanding of the source systems across sites and aligning on best practices for mapping logic can improve the reliability of the mapped data. These insights lay the groundwork for creating harmonized datasets to support robust multicenter research and clinical analytics.

## Supplementary material

10.2196/90246 Multimedia Appendix 1 SQL scripts using the vocabularies and relationships defined in the Observational Medical Outcomes Partnership Common Data Model version 5.4.

## Abbreviations

CDM: Common Data Model
DSI: Data Science Institute
DWH: Data Warehouse
EHR: electronic health record
ETL: extract, transform, and load
FHIN: Federated Health Innovation Network
*ICD-10*: *International Classification of Diseases, 10th Revision*
LOINC: Logical Observation Identifiers Names and Codes
MOC: Multidisciplinary Oncology Consultation
MZG: Minimale Ziekenhuisgegevens
OMOP: Observational Medical Outcomes Partnership
SNOMED CT: Systematized Nomenclature of Medicine–Clinical Terms
TNM: tumor, node, and metastasis
