# Supplementary material for: Addressing Data Quality Challenges in Lung Cancer Data Within the Observational Medical Outcomes Partnership Common Data Model: Observational Study
Source: J Med Internet Res. 2026 Jun 8;28:e90246. doi: 10.2196/90246 (PMC13245838; doi:10.2196/90246)
Supplement: Multimedia Appendix 1 [file jmir-v28-e90246-s001.docx]

Appendix 1

SQL script using the vocabularies and relationships as defined in OMOP-CDM V5.4:

SELECT distinct
 concat('MZG_', diagnose___code) as sourceCode,

diagnose___code as sourceName,
 1 as sourceFrequency,

COALESCE(c2.concept_id, 0) as conceptId,

CAST(NULL as varchar(255)) AS sourceAutoAssignedConceptIds,
 REPLACE(COALESCE(c2.CONCEPT_NAME, 'Unmapped'), ',', '') AS conceptName,
 (CASE WHEN CONCEPT_ID_2 IS NOT NULL

THEN 'APPROVED' ELSE 'UNCHECKED' END) AS mappingStatus,
 COALESCE(c2.DOMAIN_ID, NULL) AS domainId,
 c2.vocabulary_id AS vocabularyId,
 CAST(NULL as varchar(255)) as className,
 1 AS matchScore,
 (CASE WHEN CONCEPT_ID_2 IS NOT NULL

THEN 'EQUAL' ELSE 'UNREVIEWED' END ) AS equivalence,
 'AUTO' AS statusSetBy,
 DATEDIFF_BIG(millisecond, '1970-01-01 00:00:00', GETUTCDATE())

as statusSetOn,
 'MAPS_TO' AS mappingType,
 'AUTO' AS comment,
 'AUTO' AS createdBy,
 DATEDIFF_BIG(millisecond, '1970-01-01 00:00:00', GETUTCDATE())

as createdOn,
 CAST(NULL as varchar(255)) AS assignedReviewer
FROM fhin_raw.fhin_mzg m
INNER JOIN fhin_omop.concept AS c
 ON (CASE WHEN LEN(diagnose___code) < 4

THEN diagnose___code

ELSE CONCAT(LEFT(diagnose___code, 3), '.',

RIGHT(diagnose___code, LEN(diagnose___code) - 3)) END) = c.concept_code
INNER JOIN fhin_omop.concept_relationship AS cr
 ON cr.concept_id_1 = c.concept_id

AND cr.relationship_id = 'Maps to'
INNER JOIN fhin_omop.concept AS c2
 ON cr.concept_id_2 = c2.concept_id

AND c2.standard_concept = 'S'
WHERE
 c2.domain_id = 'Condition'

Resulting csv file (top 10 lines)

| sourceCode | sourceName | sourceFrequency | conceptId | sourceAutoAssignedConceptIds | conceptName | mappingStatus | domainId | vocabularyId | className | matchScore | equivalence | statusSetBy | statusSetOn | mappingType | comment | createdBy | createdOn | assignedReviewer |
| --- | --- | --- | --- | --- | --- | --- | --- | --- | --- | --- | --- | --- | --- | --- | --- | --- | --- | --- |
| MZG_A044 | A044 | 1 | 192815 | NULL | Intestinal infection due to E. coli | APPROVED | Condition | SNOMED | NULL | 1 | EQUAL | AUTO | 1729150789253 | MAPS_TO | AUTO | AUTO | 1729150789253 | NULL |
| MZG_A045 | A045 | 1 | 198334 | NULL | Enteric campylobacteriosis | APPROVED | Condition | SNOMED | NULL | 1 | EQUAL | AUTO | 1729150789253 | MAPS_TO | AUTO | AUTO | 1729150789253 | NULL |
| MZG_A047 | A047 | 1 | 193688 | NULL | Clostridioides difficile infection | APPROVED | Condition | SNOMED | NULL | 1 | EQUAL | AUTO | 1729150789253 | MAPS_TO | AUTO | AUTO | 1729150789253 | NULL |
| MZG_A0471 | A0471 | 1 | 4307981 | NULL | Clostridium difficile colitis | APPROVED | Condition | SNOMED | NULL | 1 | EQUAL | AUTO | 1729150789253 | MAPS_TO | AUTO | AUTO | 1729150789253 | NULL |
| MZG_A0471 | A0471 | 1 | 4323342 | NULL | Recurrent bacterial infection | APPROVED | Condition | SNOMED | NULL | 1 | EQUAL | AUTO | 1729150789253 | MAPS_TO | AUTO | AUTO | 1729150789253 | NULL |
| MZG_A0472 | A0472 | 1 | 4307981 | NULL | Clostridium difficile colitis | APPROVED | Condition | SNOMED | NULL | 1 | EQUAL | AUTO | 1729150789253 | MAPS_TO | AUTO | AUTO | 1729150789253 | NULL |
| MZG_A048 | A048 | 1 | 193402 | NULL | Bacterial enteritis of intestine | APPROVED | Condition | SNOMED | NULL | 1 | EQUAL | AUTO | 1729150789253 | MAPS_TO | AUTO | AUTO | 1729150789253 | NULL |
| MZG_A0839 | A0839 | 1 | 196620 | NULL | Viral enteritis of intestine | APPROVED | Condition | SNOMED | NULL | 1 | EQUAL | AUTO | 1729150789253 | MAPS_TO | AUTO | AUTO | 1729150789253 | NULL |
| MZG_A084 | A084 | 1 | 198678 | NULL | Intestinal infectious disease | APPROVED | Condition | SNOMED | NULL | 1 | EQUAL | AUTO | 1729150789253 | MAPS_TO | AUTO | AUTO | 1729150789253 | NULL |
| MZG_A088 | A088 | 1 | 198678 | NULL | Intestinal infectious disease | APPROVED | Condition | SNOMED | NULL | 1 | EQUAL | AUTO | 1729150789253 | MAPS_TO | AUTO | AUTO | 1729150789253 | NULL |
